# A Multi-Functional Synthetic Gene Network: A Frequency Multiplier, Oscillator and Switch

**DOI:** 10.1371/journal.pone.0016140

**Published:** 2011-02-17

**Authors:** Oliver Purcell, Mario di Bernardo, Claire S. Grierson, Nigel J. Savery

**Affiliations:** 1 Department of Engineering Mathematics, Bristol Centre for Complexity Sciences, University of Bristol, Bristol, United Kingdom; 2 Department of Engineering Mathematics, University of Bristol, Bristol, United Kingdom; 3 Department of Systems and Computer Engineering, University of Naples Federico II, Napoli, Italy; 4 School of Biological Sciences, University of Bristol, Bristol, United Kingdom; 5 School of Biochemistry, University of Bristol, Bristol, United Kingdom; Virginia Tech, United States of America

## Abstract

We present the design and analysis of a synthetic gene network that performs frequency multiplication. It takes oscillatory transcription factor concentrations, such as those produced from the currently available genetic oscillators, as an input, and produces oscillations with half the input frequency as an output. Analysis of the bifurcation structure also reveals novel, programmable multi-functionality; in addition to functioning as a frequency multiplier, the network is able to function as a switch or an oscillator, depending on the temporal nature of the input. Multi-functionality is often observed in neuronal networks, where it is suggested to allow for the efficient coordination of different responses. This network represents a significant theoretical addition that extends the capabilities of synthetic gene networks.

## Introduction

Over the past decade synthetic biologists have engineered gene networks to perform a variety of functions [1]–[3], including switches [4]–[8] and oscillators [6], [9]–[13]. Oscillators have been a focus of research and a number of examples now exist; for a review of the available synthetic oscillators see [14]. A logical and necessary next step is the development of gene networks that are capable of capturing and using the information contained in these oscillations. Frequency multiplication is one such operation; input oscillations are processed to give an oscillatory output with a multiple frequency of the input. Networks capable of performing frequency multiplication, and their various linear combinations, would allow a number of cellular processes or synthetic systems to be temporally coordinated on different time scales. This coordination could be with reference to each other, or a single ‘master clock’. A single master clock is an efficient way of ‘keeping time’ within a large network, and could be driven autonomously, or respond to external stimuli. A recently published GRN (Gene Regulatory Network) designed as a push-on push-off switch [8] displays frequency multiplication within its dynamics, but it is not designed to process an internal input, or one that is continuously oscillating. Both of these features are requirements for integration with current oscillators. We present the *in silico* design of a novel GRN capable of functioning as a frequency multiplier of one half for a continuously oscillating internal input, specifically the concentration of a transcription factor. We construct an Ordinary Differential Equation (ODE) model of the network and explain the frequency multiplier functionality through a bifurcation analysis of this model.

Lu *et al.* recently set out the challenges and goals for the next generation of synthetic gene networks [2]. A central aim was the development of networks with programmable functionality. Further analysis of the bifurcation structure reveals that our network is in fact multi-functional, where the function is programmed by the temporal characteristics of the input. In addition to acting as a frequency multiplier for an oscillating input, the network is capable of acting as a switch or an oscillator when the input is held constant between certain ranges. All three functions are available for a single set of parameters. This is a more sophisticated approach than requiring external intervention to either select functionality or tune single functions [15], [16]. Multi-functionality is a novel attribute in synthetic gene networks. Condensing multiple functions into a single network offers potential advantages for both efficiency and the coordination of separate functions.

The outline of the paper is as follows: We first discuss the network design and the conceptual basis for its function as a frequency multiplier. We then present the model used to represent the network. Simulations demonstrating the frequency multiplication behaviour follow, and a bifurcation analysis of the model is presented to explain the mathematical basis for its behaviour. The multi-functional nature of the network is then discussed and related to the bifurcation analysis. The potential for an *in vivo* implementation is then examined. It is shown that despite its size, almost the entire network can be constructed from well characterised components. Finally we discuss the utility and significance of the network.

## Results

### Network design

**Figure 1:**
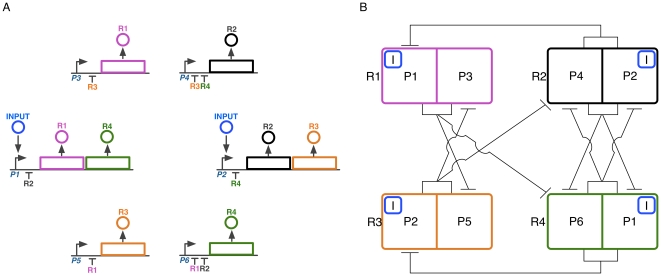
Network design. **A**. Physical representation. R1–R4 are transcriptional repressors, and P1–P6 denote promoters. ‘Input’ is a transcriptional activator. Flat-headed arrows represent repression. **B**. Node diagram representation. Each node is a repressor, divided into its two promoter sources. Input is represented by ‘I’.

The design of the network is shown in figure 1A. The network comprises 4 gene types, encoding the transcriptional repressors R1, R2, R3 and R4. Each of these genes is present in two copies, with each copy regulated by a different promoter. However, one copy each of R1 and R4 is transcribed as a single transcript, under the control of the promoter P1. Similarly, one copy each of R2 and R3 is transcribed as a single transcript under the control of promoter P2. There are six promoters (P1–P6) in total. Control of gene expression mainly occurs through repression, depicted by flat-headed arrows. Input is defined as the presence of a transcriptional activator, but could equally be the absence of a transcriptional repressor, and acts upon P1 and P2.

A node diagram of the network is shown in figure 1B. Each node is a single repressor, and the node is divided into the two promoters which control the production of each repressor's transcripts. The role of input is captured by ‘I’, attached to the promoters it acts upon. The structure and symmetries of the network are clear, for instance the mutual repression between R2 and R4, and the repression of R1 by R2 and R3 by R4.

**Figure 2:**
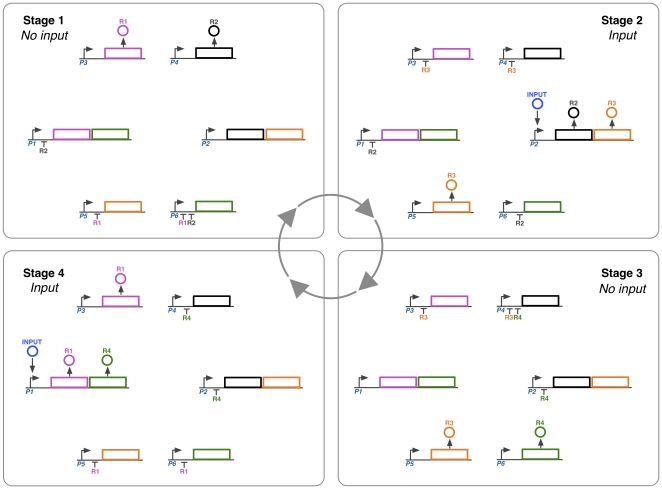
A discrete view of the frequency multiplier behaviour. The dynamics can be split into four stages, starting at stage 1 and cycling round clockwise. As the level of input switches on and off twice, each of the repressors has only made one oscillation. The network therefore functions as a frequency multiplier of one half. See main text for an explanation of the network state at each stage.

A discrete view of how the frequency multiplier behaviour arises when the input is a square wave can be seen in figure 2. The stages of the systems dynamics are as follows:

- **Stage 1**. The system is initialised with a certain level of R1 and R2 and no input. The initial R1 and R2 repress transcription from P1, P5 and P6.
- **Stage 2**. The input is applied. The presence of input causes production of R2 and R3 from the P2 promoter to occur. Repression of P1 is maintained by R2. R1 levels degrade to a level which permits production of R3 from P5.
- **Stage 3**. The input is removed (there has now been one oscillation in the input). No production of R2 or R3 from P2 now occurs, and degradation of R2 allows production of R4 from P6. R3 is maintained from P5.
- **Stage 4**. The input is applied again, activating production of R1 and R4 from P1. R4 prevents transcription from P2. R1 represses production of R3 from P5, allowing production of R1 from P3.
- **Stage 1**. The system completes a full cycle: The input is removed again (there have now been two oscillations in the input), so there is no transcription from P1. The degradation of R4 then allows production of R2 from P4. There have been two oscillations in the input concentration, but only one in each of R1, R2 R3 and R4. Hence the network has acted as a frequency multiplier of one half.

### Modelling

A model of the network was constructed to obtain a qualitative understanding of the network dynamics and assess its functionality. The network was modelled using ODEs and mass-action kinetics, with Hill functions used to represent transcriptional activation and repression. The use of ODEs allows a bifurcation analysis of the network to be performed, a powerful way of obtaining a qualitative understanding of the network behaviour. A full model comprising 12 ODEs was simplified using the quasi-steady-state assumption on mRNA levels [17], resulting in the following model equations (see File S1 for derivation):
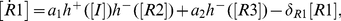
(1)

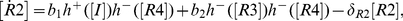
(2)

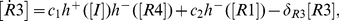
(3)

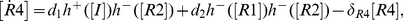
(4)


where 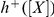 and 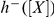 represent activating and repressing Hill functions respectively, defined as:
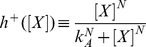
(5)

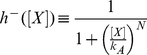
(6)


and 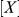 is the concentration of the specific repressor 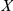, 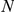 is the Hill coefficient and 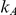 is the concentration of 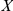 at which binding is half maximal i.e. 
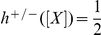
. The external input ‘I’ is the only activator within the network. 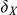 denotes the protein degradation rate for repressor 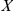. Where regulation of transcription by multiple transcriptional regulators occurs, the product is taken. This is justified from a probabilistic standpoint [18]; for transcription to occur a repressor must not be bound, and to increase the transcription rate an activator must be bound. The probability of an activator or repressor being bound and unbound respectively at any given time is defined by their respective Hill functions. Binding is assumed to be independent and therefore taking the product gives the desired probability. The parameters 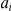, 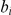, 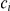 and 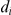, where 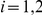 are derived in File S1. We will assume that 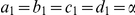, where:
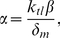
(7)


and 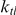 is the translation rate, 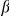 is the maximum transcription rate and 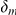 is the mRNA degradation rate. We also assume 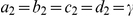, where:
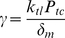
(8)


and 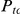 is the unrepressed transcription rate. The current model makes the assumption of zero transcription from promoters P1 and P2 in the absence of input.

For further simplicity the Hill coefficient and 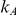 are also assumed to be the same for each Hill function. This simplified model uses 8 parameters, given in table 1. A discussion of the parameter ranges used is given in File S2.

**Table 1:** Network parameters used in simulations.

Parameter	Value	Units
Translation rate ( 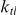 )	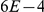	s^−1^
mRNA degradation rate ( 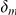 )	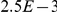	s^−1^
Protein degradation rate ( 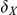 )	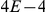	s^−1^
Hill coefficient (N)	1.3	scalar
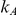 for activator	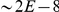	M
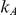 for repressor	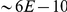	M
Maximum transcription rate (P1 and P2) ( 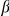 )	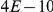	M.s^−1^
Unrepressed transcription rate (P3–P6) ( 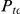 )	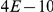	M.s^−1^

Exact values for activator and repressor 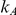 are 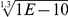 and 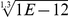 respectively.

### Frequency multiplier behaviour

**Figure 3:**
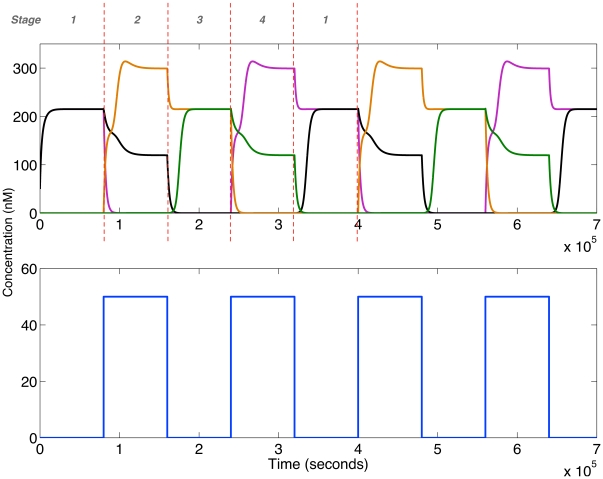
Frequency multiplication for a discrete square wave input. Time series for the repressors R1–R4 and the input are shown in the top and bottom panels respectively. The concentrations of R1, R2, R3 and R4 are represented by pink, black, orange and green lines respectively. The stages corresponding to figure 2 are shown in the top panel. Initial conditions: 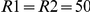 nM, 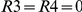 nM. Parameters from table 1 are used.

We first confirmed the discrete switching behaviour described in figure 2. Numerical simulations in figure 3 show the network performing frequency multiplication of one half on a square wave input, i.e. the period of the oscillations in R1 to R4 is twice that of the input.

**Figure 4:**
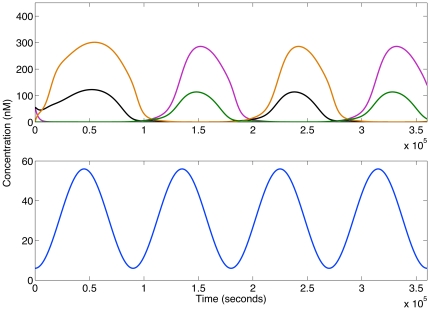
Frequency multiplication for a sine input with an offset. Time series for the repressors R1–R4 and the input are shown in the top and bottom panels respectively. The concentrations of R1, R2, R3 and R4 are represented by pink, black, orange and green lines respectively. Initial conditions: 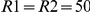 nM, 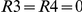 nM. The input is the following function: 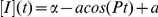, where 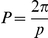, p is the period, t is time, a is amplitude and 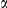 is the minimum of the input. 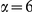 nM. Parameters from table 1 are used.

However, in order to integrate with existing genetic oscillators *in vivo* the network must be capable of performing frequency multiplication on a continuously oscillating input. Numerical simulations showed the network performing frequency multiplication of one half on an oscillating input with a period of 90000 seconds (25 hours) (see File S3). We confirmed that frequency multiplication was also possible with a slightly weaker repressor 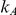 of 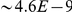 M (data not shown) (the parameters used are given in File S2). Furthermore, the oscillators constructed so far *in vivo* do not generally reach a zero level in between oscillations [14]. A frequency multiplier must therefore be capable of working with oscillations that have a non-zero minimum, or offset. Figure 4 demonstrates the network performing frequency multiplication on an oscillating input with an input minimum of 6 nM. This is approximately 10% of the maximum level, comparable to oscillations generated by the recently constructed robust oscillator in [11].

### Bifurcation analysis

In order to investigate the origin and robustness of the frequency multiplier behaviour, a bifurcation analysis of model (1)–(4) was performed under variation of a constant and then periodic input. In order to preserve correspondence to the physical system, we performed the analysis on the dimensionalised equations. The software package AUTO [19] was used to carry out all continuations.

**Figure 5:**
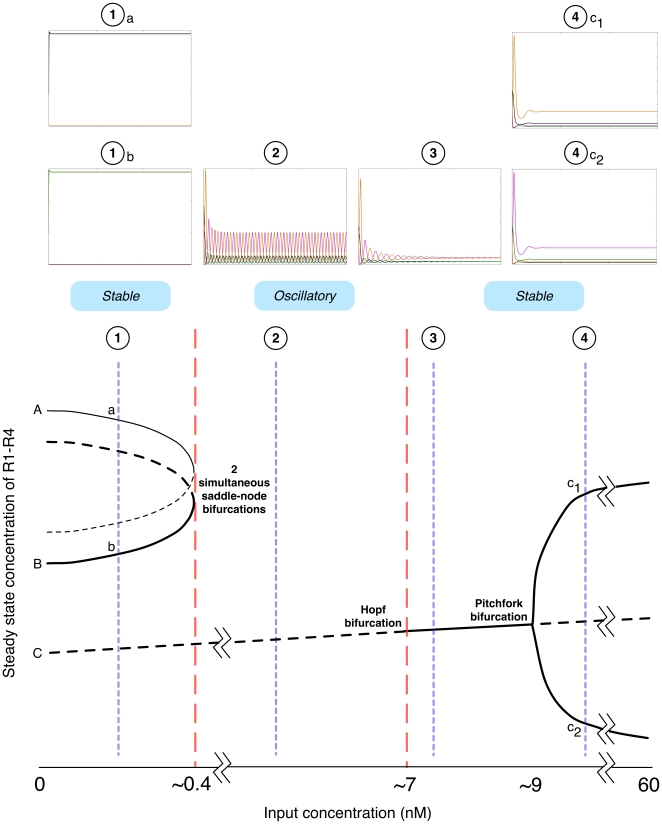
1-dimensional sketch summarising the bifurcation structure. The three main features are two simultaneous saddle-node bifurcations, a Hopf bifurcation and a pitchfork bifurcation. These occur at input concentrations of 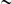
0.4 nM, 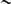
7 nM and 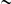
9 nM respectively. The analysis covers the input concentration range 0–60 nM, and traces out three branches of equilibria, A, B and C. The structure can be divided into four dynamical regions corresponding to labels 1–4. The dynamics at each label are shown in the set of simulations above. At position 1 and 4 two stable equilibria exist simultaneously. In all simulation panels the horizontal axis is time (seconds) and the vertical axis is concentration. All simulations are for 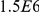 seconds. The concentration range on the vertical axis in panels 1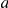 and 1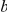, 2, 3 and 4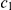 and 4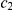, are 0–150 nM, 0–90 nM, 0–140 nM and 0–180 nM respectively. Simulation panels 4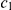 and 4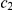 used initial conditions 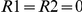 nM, 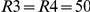 nM. All other panels used initial conditions 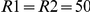 nM, 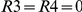 nM. Panels 1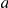 and 1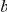, 2, 3 and 4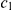 and 4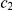 use a constant input concentration 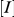 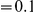 nM, 5 nM, 7.5 nM and 10 nM (4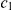 and 4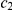) respectively. All simulations use table 1 parameters. Red dashed lines delineate the oscillatory region, which lies in between two stable regions in which trajectories decay to equilibrium levels. This diagram is intended to convey the qualitative aspects of the phase portrait. As such there is no scale on the vertical axis.

Six continuation of equilibria experiments were performed, using automatic branch switching where appropriate. Initial estimates of the model equilibria were obtained through numerical integration in MATLAB (The Mathworks, Natick, MA) and the numerical solvers in MAPLE (Maplesoft, Waterloo, ON) and are summarised in table 2. Two stable equilibria (‘
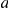
’ and ‘
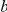
’) are characterised by zero concentrations of R1 and R2 (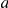) or R3 and R4 (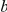) respectively, while equilibrium ‘
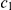
’ is characterised by low concentrations of all repressors except R3. Figure 5 depicts a 1-dimensional schematic bifurcation diagram summarising the results of all the continuation runs.

**Table 2:** Network parameters used in simulations.

Equilibrium	Associated approximate concentrations (nM) ({R1,R2,R3,R4})	Bifurcations uncovered
a. [I] = 0.1 nM	{144,144,0,0}	Saddle-node bifurcation
b. [I] = 0.1 nM	{0,0,144,144}	Saddle-node bifurcation
C_1_. [I] = 0.1 nM	{4,8,27,3}	Pitchfork and Hopf

Exact values for activator and repressor 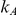 are 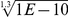 and 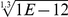 respectively.

The bifurcation structure has a number of important features. As the concentration of the input is increased we detect two simultaneous saddle-node bifurcations of equilibria 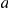 and 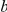 at 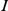 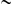
0.4 nM, leading to their disappearance. Continuation of equilibria 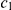 shows the occurrence of a supercritical Hopf bifurcation at 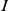 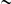
7 nM and a pitchfork bifurcation at 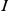 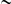
9 nM. The model behaviour can be divided into four distinct dynamical regions, corresponding to labels 1–4 in figure 5:

1. A region of coexistence of two stable and three unstable equilibria for 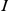 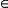 [0, 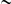
0.4].
2. A region where a single unstable equilibrium exists, together with stable undamped oscillations emerging from a Hopf bifurcation for 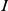 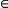 [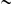
0.4, 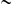
7].
3. A region where a single stable focus exists, for 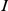 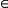 [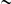
7, 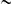
9].
4. A region of coexistence of two stable and one unstable equilibrium for 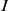 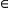 [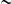
9, 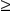
60].

**Figure 6:**
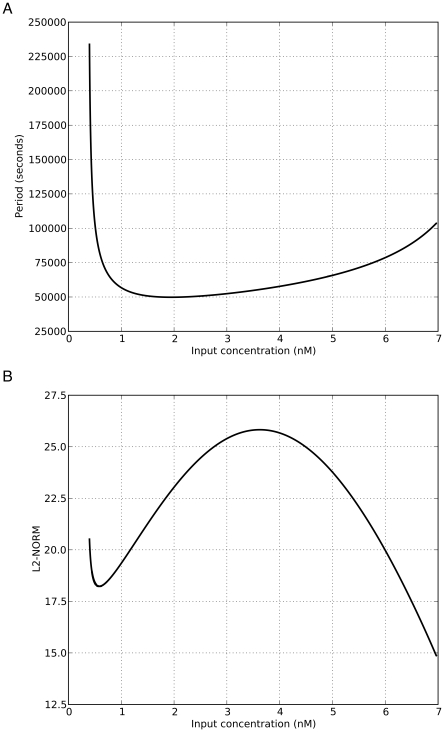
Effect of input concentration on oscillation characteristics. 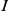
 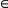 [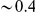, 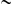
7]. **A**. Relationship between input concentration and period (seconds). **B**. Relationship between input concentration and the L2-norm (in this case the norm of a vector representing the amplitude of R1 to R4).

If the input concentration is held constant, such that the system is in the region of the bifurcation structure between the saddle-node bifurcation and the Hopf bifurcation, a stable limit cycle exists and the dynamics are oscillatory. Figures 6A and 6B show how the oscillation period and amplitude change over the 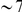 nM range where oscillations are observed. The most notable feature is the near-vertical increase in oscillation period as the input approaches the concentration at which the saddle-node bifurcations occur. This suggests that an infinite-period bifurcation takes place [20].

To investigate the frequency multiplier behaviour, continuation of limit cycles of a periodically forced system was performed on a modified set of equations with the oscillating input (forcing) defined autonomously (see File S4). Numerical time series data describing a single period of a period-1 solution of the modified system of equations was used as an initial condition for continuation. Continuation was performed using the period of the input as the bifurcation parameter (the amplitude of the input was 50 nM as in simulations).

**Figure 7:**
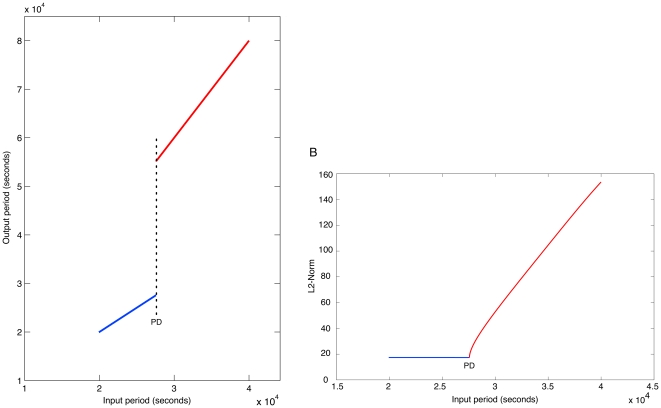
Existence of a period-doubling bifurcation. A period-doubling is observed at an input period of 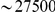 seconds (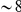 hours). The period-doubling point is labelled as ‘PD’. Blue lines and red lines represent period-1 and period-2 solutions respectively. **A**. Relationship between input period and ‘output’ period, where output denotes the proteins R1 to R4. **B**. Relationship between input period and L2-Norm.

Continuations demonstrate that the frequency multiplier functionality is a consequence of a period-doubling bifurcation as the period of the input crosses a certain threshold. Figure 7A shows the period of the ‘output’ i.e. proteins R1 to R4, as a function of the input period. Prior to the period-doubling bifurcation the output period is equal to the input period (blue line). A period-doubling bifurcation occurs at 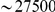 seconds (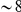 hours), after which (red line) the output period is twice the period of the input. Equivalently the output frequency is half the input frequency. The existence of a period doubling bifurcation is further confirmed in figure 7B, which shows the relationship between the input period, and the L2-Norm. As expected for a period-doubling bifurcation, the L2-Norm of the period-2 limit cycle is equal to the period-1 limit cycle at the bifurcation point.

This result shows that for the parameter values in table 1, frequency multiplication can be performed on a wide range of input periods, from 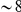 hours (figure 7A) to at least 140 hours (data not shown). Our network is therefore theoretically capable of interfacing with existing long-period oscillators [6], [13]. The majority of currently available oscillators exhibit oscillations with periods shorter than 8 hours [14], and frequency multiplication on these high frequency oscillators may be possible under different parameter regimes.

### Multi-functionality

To date, synthetic gene networks have possessed single functions. The preceding investigation of the bifurcation structure suggests that our network possesses other functions in addition to frequency multiplication. Specifically, the network is also capable of functioning as an oscillator or a switch.

The oscillatory behaviour in figure 5 demonstrates that, if the input concentration is held constant within a certain range (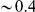 to 7 nM), the network functions as an oscillator. While the explanation of the oscillator function is straightforward from a mathematical perspective, it is decidedly more complicated in terms of gene interactions, but can be understood by examining simulations of the full model (data not shown). The steps of a single cycle are described sequentially below.

1. The system is initialised with some R1 and R2.
2. Depending on their initial concentrations, R1 and R2 concentrations increase due to production from P3 and P4 as there is no R3 or R4 present within the system.
3. Although the input is positive, there is no production from P1 as R2 is present. However R3 increases from P2 and switches off production of R1 and R2 from P3 and P4 respectively. This state is similar to stage 1 of figure 2.
4. This causes R1 and R2 levels to drop. R1 drops rapidly as P3 was its only source. R2 drops more slowly as it is still produced from P2.
5. R3 from P5 increases as R1 is its only repressor. This state is similar to stage 2 of figure 2.
6. Although there is repression of P6 from P2 R2, a **positive feedback** loop is formed, whereby R4 from P6 represses P2, reducing the level of R2, which reduces repression on P6, further increasing R4 and repressing P2, and so on. As such R2 decreases and R4 from P6 increases, toward some equilibrium.
7. R2 is now at a low level. This allows input to switch on P1 and increase R1 and R4, causing R3 and R4 from P5 and P6 respectively to be repressed.
8. R1 then increases rapidly, as with low R3, production also increases through P3.
9. R2 increases from P4 with another **positive feedback** loop existing between P4 and P1 whereby R2 switches off P1 which decreases R4 which allows further R2 increase, and so on, again toward some equilibrium. This increase in R1 from P3 and R2 from P4 brings the system back toward stage 1, completing a cycle.

At a low constant level of input, below the oscillatory range, the system remains at step 3 as the level of input is not high enough to produce enough R3 from P2 to significantly repress P3 and P4 and move the system onto step 4. Alternatively, at a high constant level of input, above the oscillatory range, the system remains at step 5 as there is enough R2 being produced from P2 to maintain repression of R4 production from P1 and P6, which prevents the positive feedback loop in step 6 from occurring. At an intermediate constant input, within the oscillatory range of figure 5, the level of input is high enough to move the system on from step 3 to 4, but low enough to allow progression from step 5 to 6. The system can then freely progress through step 1 to 9 sequentially, generating oscillatory dynamics.

**Figure 8:**
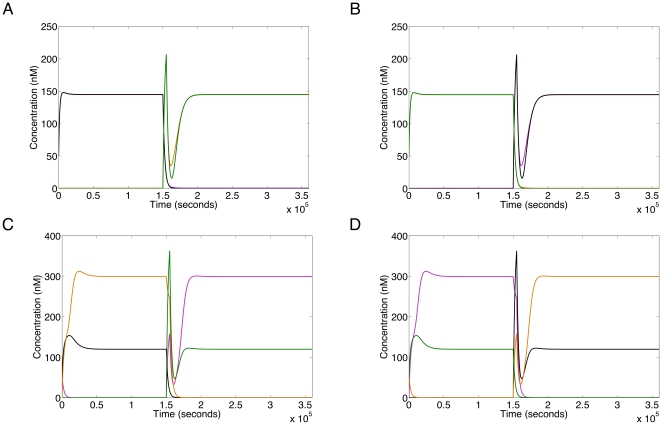
Demonstration of switch function. In each case the system is allowed to reach equilibrium under a constant level of input. The concentrations of R1, R2, R3 and R4 are represented by pink, black, orange and green lines respectively. **A**. Switch from [R1 & R2 high, R3 & R4 low] to [R3 & R4 high, R1 & R2 low], at an input of 0.1 nM. **B**. Switch from [R3 & R4 high, R1 & R2 low] to [R1 & R2 high to R3 & R4 low], at an input of 0.1 nM. **C**. Switch from [R2 & R3 high, R1 & R4 low] to [R1 & R4 high, R2 & R3 low], at an input of 50 nM. **D**. Switch from [R1 & R4 high, R2 & R3 low] to [R2 & R3 high, R1 & R4 low], at an input of 50 nM. In **A** and **C** the switch is performed by increasing the 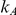 for R1 and R2 binding from 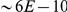 M to 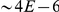 M (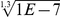 exactly) between the times 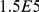 and 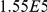 seconds. In **B** and **D** the switch is performed by increasing the 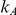 for R3 and R4 binding by the same amount and duration. Initial conditions of 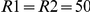 nM, 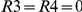 nM for **A** and **C**, and 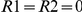 nM, 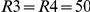 nM for **B** and **D**. Parameters from table 1 are used.

The bifurcation structure also reveals that if the input is held constant between either the concentrations 0 to 0.4 nM or 9 to 60 nM, the network exhibits bi-stability. This allows the network to function as a toggle switch if the binding affinity of particular repressors is temporarily lowered. This can be done *in vivo* by small molecules termed ‘inducers’ [4]. This toggle switch behaviour can be achieved for a very low concentration (figures 8A and 8B) and a high concentration (figures 8C and 8D). It is likely that switching can be achieved for a range of constant input values far exceeding 60 nM. If we consider the network within *E. coli*, one can use the approximation that 1 molecule corresponds to a concentration of 1 nM. Then the low input range for switching is probably physically irrelevant as the concentration corresponds to less that a single molecule. However, in cells with larger volumes these lower concentrations will become more relevant.

In summary, the three functions of the network are:

1. **Frequency multiplier of one half**. When the input to the network is oscillatory the output of the network is oscillatory with a frequency half that of the input.
2. **Oscillator**. When the input is maintained within a certain range the network oscillates.
3. **Switch**. When the input is maintained within a certain range, external modulation of particular repressive strengths allows switching between different steady-state protein levels.

### Prospects for *in vivo* implementation

The model uses symmetric parameters. However, some asymmetry will inevitably exist in an *in vivo* implementation, even with well matched components. We therefore used numerical simulations to investigate the effect of various sample forms of asymmetry on each of the three functions. In each simulation the value of a single parameter was changed from the value stated in table 1, whilst all other parameters were kept at the values stated in table 1. We found that all three functions were robust to at least some forms of asymmetry (see File S5), and we therefore conclude that the network's functions do not depend on the use of a completely symmetrical parameter set. The failure to observe functionality in some of our asymmetric simulations does not necessarily indicate that the parameters in question must be symmetrical in order for the network to function, as it might be possible to restore functionality by compensatory changes in other parameters in the network. A much fuller description of parameter space is required to properly understand the parameter dependence of the three functions, and would direct the choice of components used in a *in vivo* implementation.

Intrinsic noise is another physical reality that must be considered. Stochastic simulations in the form of Chemical Langevin Equations (CLEs) were performed to assess the robustness of the three functions to noise (parameters from table 1 used). In each case the function was observed (see File S6), demonstrating the function is robust to intrinsic noise.

**Figure 9:**
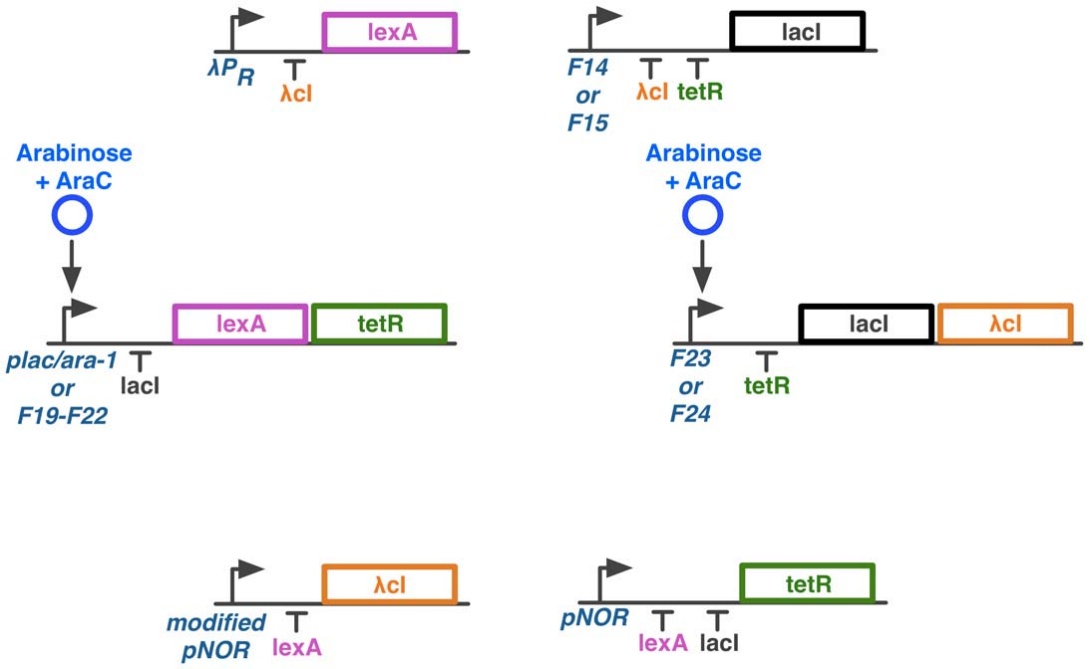
Possible *in vivo* implementation of the network. The repressors used are the set 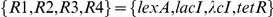. The promoters used are: Fx e.g. F23 [21], P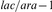 [35], pNOR [8] and 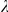
P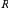. Modified pNOR is the pNOR promoter with repression by LacI removed. The input to the network is arabinose + AraC, which form a complex that can activate transcription.

A possible implementation of the network is given in figure 9. It is based on constructing the network in *E. coli*, a tried and tested host for synthetic networks [4], [6], [9]–[11].

The implementation proposed here uses the bacterial transcription repressors LexA, LacI, 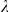
cI and TetR, and the bacterial transcriptional activator AraC. These have all been used before in the construction of synthetic networks [4], [6], [8], [9], [11], as have the promoters P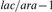 [11], 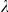
P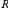 [9] and pNOR [8]. The promoters Fx have all been constructed and characterised [21], but the modified pNOR would require construction and characterisation. Arabinose, which binds to AraC allowing it to activate transcription, has also been previously used [11]. However, in this implementation it would align better with modelling if Arabinose was maintained at a saturating level, and AraC used as the varying input.

Despite the availability of components, a functional *in vivo* construction of the network poses two main challenges. Firstly, none of the promoters so far used in the construction of synthetic networks have been characterised for control by an externally controlled oscillating input. Methods for controlling gene expression, such as an oscillating arabinose concentration (figure 9), light [22] and temperature [23] could be explored, as these are capable of regulating gene expression in a reversible manner. Secondly, even if the network was shown to be theoretically compatible with the output from an existing oscillator, the repertoire of repressors that have been used and characterised in the context of synthetic networks is not extensive enough to allow for construction of both networks without an overlapping use of components. Other naturally occurring repressors could be used, but these are untested and add more uncertainty to the construction. Ultimately, the design and construction of large networks with functions only available within specific parameter regimes will require libraries of well characterised artificial transcription factors [24]–[27] that are orthogonal to the host system.

## Discussion

A theoretical synthetic gene regulatory network has been presented, and a possible *in vivo* implementation discussed. The network is capable of performing frequency multiplication of one half on an oscillating transcription factor concentration, such as those produced by the currently available oscillators [14]. The network takes an oscillating transcription factor concentration as an input, and produces an oscillatory output with half the frequency of the input, in the form of the concentration of a transcriptional repressor. Bifurcation analysis demonstrates that the frequency multiplier functionality is a result of a period-doubling bifurcation. This is the first synthetic gene network presented that is theoretically capable of performing frequency multiplication on a continuously oscillating input from an intracellular source. The development of genetic frequency multipliers could allow cellular processes and synthetic systems existing on various different time scales to be temporally coordinated, either with reference to each other, or a single ‘master clock’ that keeps time for the network.

Bifurcation analysis reveals that the network is multi-functional. For a single set of parameters the network can display one of three functions, which can be selected by changing the temporal characteristics of the input. Parallels can be drawn with the field of neuroscience where multi-functional circuits are widespread in both invertebrates and vertebrates [28]–[31] (see [32] for a recent review). Multi-functionality has also recently been observed on a basic level in both coherent and incoherent feed-forward-loops [33], [34], which are small regulatory motifs present in some gene and protein networks.

The obvious benefit of using multi-functional networks is efficiency; network components are re-used under different dynamic, functional regimes [32]. This allows a number of single function networks to be condensed into one multi-functional network. It is also proposed that multi-functional networks allow functions to be better coordinated. In the marine mollusk *Tritonia*, crawling, withdrawal and swimming are all part of the escape response. The use of a single multi-functional neural network ensures this vital response is correctly temporally integrated [31]. Multi-functionality is not a property often ascribed to natural gene networks, probably because natural networks are rarely fully characterised and inputs and outputs are difficult to define. Despite this, the size and interconnectivity of natural networks may mean that multi-functionality can readily be found at some level. Indeed, the work in neural systems demonstrates that multi-functionality is a property selected for in evolution, and therefore would be expected to arise in other biological contexts.

The single-function synthetic networks considered in the literature so far have been rationally designed, with larger networks set to be constructed in a modular fashion by connecting up these networks [8]. Of the three functions of our network, only frequency multiplication was rationally designed, while the switch and oscillator functions were discovered. In particular, the oscillator function is difficult to understand in terms of gene-gene interactions, and would arguably be impossible to rationally design using current methodologies. This work suggests that while multi-functionality may be difficult to design on purpose, it may be difficult to avoid by chance, and may be a property that emerges increasing frequently as synthetic networks become larger and more complex. The challenge will then be to ensure that the design process identifies and exploits any benefits that multi-functionality brings if and when it arises.

The network presented here is novel in two respects. Firstly, it is capable of performing frequency multiplication on a sinusoidally oscillating input, suggesting it is capable of integrating with current oscillators. Secondly, it possesses programmable multi-functionality, specifically the ability to select between one of three functions by changing the nature of the input. Collectively, this network represents a significant theoretical addition to the capabilities of current synthetic gene networks.

## Materials and Methods

Numerical simulations were performed using custom MATLAB (The Mathworks, Natick, MA) scripts and the ode45 numerical integrator. Stochastic simulations used the fixed step numerical integrator ode4. Continuations of both equilibria and limit cycles were performed in AUTO 07p [19], using the constant input level and period of input as bifurcation parameters respectively. Ranges for steady-state values of the system for a constant level of input were obtained by simulation in MATLAB (The Mathworks, Natick, MA). MAPLE (Maplesoft, Waterloo, ON) was then used to numerically solve within the range to obtain steady-state values with a precision adequate for starting continuations.

## Supporting Information

File S1 **Model derivation.**
(PDF)

File S2 **Model parameterisation.**
(PDF)

File S3 **Frequency multiplication for an oscillating input returning to zero.**
(PDF)

File S4 **Continuation of limit cycles.**
(PDF)

File S5 **Simulations investigating parameter asymmetry.**
(PDF)

File S6 **Stochastic simulations.**
(PDF)

## References

[1] Purnick PEM, Weiss R (2009). The second wave of synthetic biology: from modules to systems.. Nat Rev Mol Cell Biol.

[2] Lu TK, Khalil AS, Collins JJ (2009). Next-generation synthetic gene networks.. Nat Biotech.

[3] Khalil AS, Collins JJ (2010). Synthetic biology: applications come of age.. Nat Rev Genet.

[4] Gardner TS, Cantor CR, Collins JJ (2000). Construction of a genetic toggle switch in Escherichia coli.. Nature.

[5] Kramer BP, Viretta AU, Daoud-El-Baba M, Aubel D, Weber W (2004). An engineered epigenetic transgene switch in mammalian cells.. Nat Biotech.

[6] Atkinson MR, Savageau MA, Myers JT, Ninfa AJ (2003). Development of genetic circuitry exhibiting toggle switch or oscillatory behavior in Escherichia coli.. Cell.

[7] Deans TL, Cantor CR, Collins JJ (2007). A tunable genetic switch based on RNAi and repressor proteins for regulating gene expression in mammalian cells.. Cell.

[8] Lou C, Liu X, Ni M, Huang Y, Huang Q (2010). Synthesizing a novel genetic sequential logic circuit: a push-on push-off switch.. Mol Syst Biol.

[9] Elowitz MB, Leibler S (2000). A synthetic oscillatory network of transcriptional regulators.. Nature.

[10] Fung E, Wong WW, Suen JK, Bulter T, Lee S (2005). A synthetic gene-metabolic oscillator.. Nature.

[11] Stricker J, Cookson S, Bennett MR, Mather WH, Tsimring LS (2008). A fast, robust and tunable synthetic gene oscillator.. Nature.

[12] Tigges M, Marquez-Lago TT, Stelling J, Fussenegger M (2009). A tunable synthetic mammalian oscillator.. Nature.

[13] Tigges M, Dénervaud N, Greber D, Stelling J, Fussenegger M (2010). A synthetic low-frequency mammalian oscillator.. Nucleic Acids Res.

[14] Purcell O, Savery NJ, Grierson CS, di Bernardo M (2010). A comparative analysis of synthetic genetic oscillators.. J R Soc Interface.

[15] Sohka T, Heins RA, Phelan RM, Greisler JM, Townsend CA (2009). An externally tunable bacterial band-pass filter.. Proc Natl Acad Sci USA.

[16] Conrad E, Mayo AE, Ninfa AJ, Forger DB (2008). Rate constants rather than biochemical mechanism determine behaviour of genetic clocks.. J R Soc Interface.

[17] Polynikis A, Hogan SJ, di Bernardo M (2009). Comparing different ODE modelling approaches for gene regulatory networks.. Journal of Theoretical Biology.

[18] Alon U (2007). An Introduction to Systems Biology: Design Principles of Biological Circuits..

[19] Doedel E (2007). AUTO 07..

[20] Kuznetsov YA (2004). Elements of Applied Bifurcation Theory (3rd Edition)..

[21] Kinkhabwala A, Guet CC (2008). Uncovering cis regulatory codes using synthetic promoter shuffling.. PLoS ONE.

[22] Deiters A (2009). Light activation as a method of regulating and studying gene expression.. Curr Opin Chem Biol.

[23] Neupert J, Karcher D, Bock R (2008). Design of simple synthetic RNA thermometers for temperature-controlled gene expression in Escherichia coli.. Nucleic Acids Res.

[24] Lee JY, Sung BH, Yu BJ, Lee JH, Lee SH (2008). Phenotypic engineering by reprogramming gene transcription using novel artificial transcription factors in Escherichia coli.. Nucleic Acids Res.

[25] Gommans WM, Haisma HJ, Rots MG (2005). Engineering zinc finger protein transcription factors: the therapeutic relevance of switching endogenous gene expression on or off at command.. Journal of Molecular Biology.

[26] Park KS, Jang YS, Lee H, Kim JS (2005). Phenotypic alteration and target gene identification using combinatorial libraries of zinc finger proteins in prokaryotic cells.. Journal of Bacteriology.

[27] Giesecke AV, Fang R, Joung JK (2006). Synthetic protein-protein interaction domains created by shuffling cys2his2 zinc-fingers.. Mol Syst Biol.

[28] Soffe SR (1997). The pattern of sensory discharge can determine the motor response in young Xenopus tadpoles.. J Comp Physiol A.

[29] Getting PA, Deken MS (1985). Model Neural Networks and Behavior..

[30] Getting PA (1989). Emerging principles governing the operation of neural networks.. Annu Rev Neurosci.

[31] Popescu IR, Frost WN (2002). Highly dissimilar behaviors mediated by a multifunctional network in the marine mollusk Tritonia diomedea.. J Neurosci.

[32] Briggman KL, Kristan WB (2008). Multifunctional pattern-generating circuits.. Annu Rev Neurosci.

[33] Tyson JJ, Novák B (2010). Functional motifs in biochemical reaction networks.. Annu Rev Phys Chem.

[34] Guantes R, Estrada J, Poyatos JF (2010). Trade-offs and noise tolerance in signal detection by genetic circuits.. PLoS ONE.

[35] Lutz R, Bujard H (1997). Independent and tight regulation of transcriptional units in Escherichia coli via the LacR/O, the TetR/O and AraC/I1-I2 regulatory elements.. Nucleic Acids Res.

