# Supplementary material for: A Multi-Functional Synthetic Gene Network: A Frequency Multiplier, Oscillator and Switch
Source: PLoS One. 2011 Feb 17;6(2):e16140. doi: 10.1371/journal.pone.0016140 (PMC3040778; doi:10.1371/journal.pone.0016140)
Supplement: File S1 — Model derivation. (PDF) [file pone.0016140.s001.pdf]

Supporting Information S1

Model derivation

The equations for the entire system can be written as follows:

The equations for the mRNAs are:

$$
\begin{aligned}
\frac{d[mR1P1]}{dt} &= \left(\frac{\beta_{P1}[I]^{N_{aP1}}}{k_{aP1}+[I]^{N_{aP1}}}\right)\left(\frac{1}{1+\left(\frac{[R2]^{N_{rP1}}}{k_{rP1}}\right)}\right) - \delta_{m14,1}[mR1P1], \qquad (1)\\
\frac{d[mR4P1]}{dt} &= \left(\frac{\beta_{P1}[I]^{N_{aP1}}}{k_{aP1}+[I]^{N_{aP1}}}\right)\left(\frac{1}{1+\left(\frac{[R2]^{N_{rP1}}}{k_{rP1}}\right)}\right) - \delta_{m14,1}[mR4P1],\\
\frac{d[mR2P2]}{dt} &= \left(\frac{\beta_{P2}[I]^{N_{aP2}}}{k_{aP2}+[I]^{N_{aP2}}}\right)\left(\frac{1}{1+\left(\frac{[R4]^{N_{rP2}}}{k_{rP2}}\right)}\right) - \delta_{m23,2}[mR2P2],\\
\frac{d[mR3P2]}{dt} &= \left(\frac{\beta_{P2}[I]^{N_{aP2}}}{k_{aP2}+[I]^{N_{aP2}}}\right)\left(\frac{1}{1+\left(\frac{[R4]^{N_{rP2}}}{k_{rP2}}\right)}\right) - \delta_{m23,2}[mR3P2],\\
\frac{d[mR1P3]}{dt} &= P3_{tc}\left(\frac{1}{1+\left(\frac{[R3]^{N_{rP3}}}{k_{rP3}}\right)}\right) - \delta_{m1,3}[mR1P3], \qquad (2)\\
\frac{d[mR2P4]}{dt} &= P4_{tc}\left(\frac{1}{1+\left(\frac{[R3]^{N_{r3,4}}}{k_{r3,4}}\right)}\right)\left(\frac{1}{1+\left(\frac{[R4]^{N_{r4,4}}}{k_{r4,4}}\right)}\right) - \delta_{m2,4}[mR2P4],\\
\frac{d[mR3P5]}{dt} &= P5_{tc}\left(\frac{1}{1+\left(\frac{[R1]^{N_{rP5}}}{k_{rP5}}\right)}\right) - \delta_{m3,5}[mR3P5],\\
\frac{d[mR4P6]}{dt} &= P6_{tc}\left(\frac{1}{1+\left(\frac{[R1]^{N_{r1,6}}}{k_{r1,6}}\right)}\right)\left(\frac{1}{1+\left(\frac{[R2]^{N_{r2,6}}}{k_{r2,6}}\right)}\right) - \delta_{m4,6}[mR4P6],
\end{aligned}
$$

The equations for the proteins are:

$$\frac{d[R1]}{dt} = k_{tl}[mR1] - \delta_{R1}[R1], \tag{3}$$

$$\frac{d[R2]}{dt} = k_{tl}[mR2] - \delta_{R2}[R2],$$

$$\frac{d[R3]}{dt} = k_{tl}[mR3] - \delta_{R3}[R3],$$

$$\frac{d[R4]}{dt} = k_{tl}[mR4] - \delta_{R4}[R4], \tag{4}$$

where for instance $[mR1]$ is the total concentration of R1 mRNA. In this case it has two contributions, from the P1 and P3 promoter driven genes. $[I]$ is the concentration of input and for example $N_{r3,4}$ is the Hill co-efficient for R3 acting on the P4 promoter, $\delta_{m14,1}$ is the degradation rate for the mRNAs for R1 and R4 from the P1 promoter controlled gene and $k_{r2,6}$ is the binding affinity of R2 to the promoter P6. For clarity the affinity used is $k_d = k_A^N$. $\beta_{P1/2}$ are the maximum transcription rates from promoters P1 and P2, and for instance $P3_{tc}$ is the unrepressed transcription rate from promoter P3. Hill functions, and their products, are used to describe the relationship between the concentration of a transcriptional activator and the rate of transcription (see main text).

A common way of reducing the dimensionality of gene network models is to assume that the mRNA reaches equilibrium instantaneously for a change in the relevant transcription factor concentration [1]. Setting the derivative to zero and solving for the mRNA concentration gives an algebraic relationship for the mRNA. This can then be substituted in the ODEs describing the changes in proteins. Such a simplification allows the dimensionality to be significantly reduced, while at the same time often maintaining the qualitative dynamics of the larger system. There is however no biological justification for this simplification, and in certain cases the qualitative dynamics are significantly affected [1][2]. However, it can be easily verified whether the two models agree by simply performing simulations under both for identical parameter values.

The equilibrium assumption for $[mR1]$ can be made by making an equilibrium assumption on $[mR1P1]$ and $[mR1P3]$ i.e. by setting the derivatives in equations 1 and 2 to zero, and making $[mR1P1]$ and $[mR1P3]$ the subjects, respectively. As stated, these can then be summed to find $[mR1]$.

Setting the derivative to zero in equation (1):

$$0 = \left(\frac{\beta_{P1}[I]^{N_{aP1}}}{k_{aP1} + [I]^{N_{aP1}}}\right)\left(\frac{1}{1 + \left(\frac{[R2]^{N_{rP1}}}{k_{rP1}}\right)}\right) - \delta_{m14,1}[mR1P1]. \tag{5}$$

Solving for $[mR1P1]$, we get:

$$[mR1P1] = \frac{1}{\delta_{m14,1}}\left(\frac{\beta_{P1}[I]^{N_{aP1}}}{k_{aP1} + [I]^{N_{aP1}}}\right)\left(\frac{1}{1 + \left(\frac{[R2]^{N_{rP1}}}{k_{rP1}}\right)}\right). \tag{6}$$

The ODEs describing protein dynamics require the total levels of each type of mRNA. As each mRNA has contributions from two sources, the same instantaneous equilibrium assumption needs to be made for both of these sources. This gives two algebraic relationships, one for each mRNA source. These can then be summed to give the total concentration of the mRNA.

If the equilibrium assumption is made for the other source of R1 mRNA i.e. $[mR1P3]$, this can then be added to equation (6) to give the term for $[mR1]$:

$$[mR1] = \frac{1}{\delta_{m14,1}}\left(\frac{\beta_{P1}[I]^{N_{aP1}}}{k_{aP1}+[I]^{N_{aP1}}}\right)\left(\frac{1}{1+\left(\frac{[R2]^{N_{rP1}}}{k_{rP1}}\right)}\right)+\frac{P3_{tc}}{\delta_{m1,3}}\left(\frac{1}{1+\left(\frac{[R3]^{N_{rP3}}}{k_{rP3}}\right)}\right) \tag{7}$$

This can then be substituted into equation (3) which gives the following.

$$\frac{d[R1]}{dt} = \frac{k_{tl}}{\delta_{m14,1}}\left(\frac{\beta_{P1}[I]^{N_{aP1}}}{k_{aP1}+[I]^{N_{aP1}}}\right)\left(\frac{1}{1+\left(\frac{[R2]^{N_{rP1}}}{k_{rP1}}\right)}\right)+\frac{k_{tl}P3_{tc}}{\delta_{m1,3}}\left(\frac{1}{1+\left(\frac{[R3]^{N_{rP3}}}{k_{rP3}}\right)}\right)-\delta_{R1}[R1] \tag{8}$$

If this is done for all proteins, the model can be reduced to the following four ODEs.

$$\frac{d[R1]}{dt} = \frac{k_{tl}\beta_{P1}}{\delta_{m14,1}}\left(\frac{[I]^{N_{aP1}}}{k_{aP1}+[I]^{N_{aP1}}}\right)\left(\frac{1}{1+\left(\frac{[R2]^{N_{rP1}}}{k_{rP1}}\right)}\right)+\frac{k_{tl}P3_{tc}}{\delta_{m1,3}}\left(\frac{1}{1+\left(\frac{[R3]^{N_{rP3}}}{k_{rP3}}\right)}\right)-\delta_{R1}[R1], \tag{9}$$

$$\frac{d[R2]}{dt} = \frac{k_{tl}\beta_{P2}}{\delta_{m23,2}}\left(\frac{[I]^{N_{aP2}}}{k_{aP2}+[I]^{N_{aP2}}}\right)\left(\frac{1}{1+\left(\frac{[R4]^{N_{rP2}}}{k_{rP2}}\right)}\right)+\frac{k_{tl}P4_{tc}}{\delta_{m2,4}}\left(\frac{1}{1+\left(\frac{[R3]^{N_{r3,4}}}{k_{r3,4}}\right)}\right)\left(\frac{1}{1+\left(\frac{[R4]^{N_{r4,4}}}{k_{r4,4}}\right)}\right)-\delta_{R2}[R2], \tag{10}$$

$$\frac{d[R3]}{dt} = \frac{k_{tl}\beta_{P2}}{\delta_{m23,2}}\left(\frac{[I]^{N_{aP2}}}{k_{aP2}+[I]^{N_{aP2}}}\right)\left(\frac{1}{1+\left(\frac{[R4]^{N_{rP2}}}{k_{rP2}}\right)}\right)+\frac{k_{tl}P5_{tc}}{\delta_{m3,5}}\left(\frac{1}{1+\left(\frac{[R1]^{N_{rP5}}}{k_{rP5}}\right)}\right)-\delta_{R3}[R3], \tag{11}$$

$$\frac{d[R4]}{dt} = \frac{k_{tl}\beta_{P1}}{\delta_{m14,1}}\left(\frac{[I]^{N_{aP1}}}{k_{aP1}+[I]^{N_{aP1}}}\right)\left(\frac{1}{1+\left(\frac{[R2]^{N_{rP1}}}{k_{rP1}}\right)}\right)+\frac{k_{tl}P6_{tc}}{\delta_{m4,6}}\left(\frac{1}{1+\left(\frac{[R1]^{N_{r1,6}}}{k_{r1,6}}\right)}\right)\left(\frac{1}{1+\left(\frac{[R2]^{N_{r2,6}}}{k_{r2,6}}\right)}\right)-\delta_{R4}[R4], \tag{12}$$

where the maximum transcription rates β have been taken out of the Hill functions. By representing the activating Hill functions as $h^+(X)$ and the repressive Hill functions as $h^-(X)$ where X is the relevant repressor, the equations given in the main text are produced. In reality the translation rate k_{tl} would be different, meaning that each coefficient given in the main text i.e. a_i, b_i, c_i and d_i, where $i = 1, 2$.

References

[1] Polynikis A, Hogan SJ, di Bernardo M (2009) Comparing different ODE modelling approaches for gene regulatory networks. Journal of Theoretical Biology 261: 511–30.

[2] Hilborn RC, Erwin JD (2008) Stochastic coherence in an oscillatory gene circuit model. Journal of Theoretical Biology 253: 349–54.
