# Supplementary material for: A Multi-Functional Synthetic Gene Network: A Frequency Multiplier, Oscillator and Switch
Source: PLoS One. 2011 Feb 17;6(2):e16140. doi: 10.1371/journal.pone.0016140 (PMC3040778; doi:10.1371/journal.pone.0016140)
Supplement: File S2 — Model parameterisation. (PDF) [file pone.0016140.s002.pdf]

Supporting Information S2

Model parameterisation

Seven parameter ranges are required; translation rate, mRNA degradation rate, protein degradation rate, binding co-efficient (k_A), Hill-coefficient, maximum transcription rate and basal transcription rate. Parameters will vary between different systems and experimental conditions, and the ranges here are just intended to be over plausible orders of magnitude.

- Translation rate: The range for translation rate was set as $1.67E-4\mathrm{s}^{-1}$ - $1.17E-3\mathrm{s}^{-1}$. This was taken as a range around values used in the supplementary information from [1], which were on the order of $1E-4$.
- mRNA degradation rate: In [2] it is shown that about 80% of 4,288 transcripts analysed in *E. coli* have half-lives of between 3 and 8 minutes. Using the relationship $k = \frac{ln(2)}{t_{1/2}}$ for a first-order decay equation, gives a range of $1.4E-3\mathrm{s}^{-1}$ - $3.85E-3\mathrm{s}^{-1}$
- Protein degradation: From [3], we found that half-lives for the λ transcriptional repressor DNA-binding domain are 60 minutes, and 4 minutes with a specific sequence (termed an ssrA tag) at the carboxyl end of the protein, that targets it for destruction by native *E. coli* proteases. This is used as the range, with the same relationship as for mRNA degradation rates used to convert to rate constants.
- The binding co-efficient of the transcription factor to the DNA, k_A: The ranges of values given in [4], [5] and [6] suggest a range of $1E-10\mathrm{M}$ - $5E-8\mathrm{M}$.
- Hill co-efficient: Between 1 and 2, fractions allowed.
- Maximum transcription rate: Limited by the maximum rate at which RNA polymerase can undergo promoter clearance (approximately 1 per second [7]). The bottom rate is set at one transcription every 20 minutes, the approximate doubling time of an *E. coli* growing in preferential conditions. If one uses the relationship that in the average *E. coli*, one molecule approximately corresponds to a concentration of 1 nM, then this range is 0.00083 $\mathrm{nM.s}^{-1}$ - 1 $\mathrm{nM.s}^{-1}$
- Unrepressed transcription rate: Taken as the same as the range for the maximum transcription rate.

These ranges are summarised in table S1. Table S2 gives the parameters used for the investigation of the frequency multiplier function with a weaker repressor binding affinity.

References

[1] Tigges M, Marquez-Lago TT, Stelling J, Fussenegger M (2009) A tunable synthetic mammalian oscillator. Nature 457: 309–12.

Parameter	Range
translation rate	$1.67E-4$ s^{-1} - $1.17E-3$ s^{-1}
mRNA degradation rate	$1.4E-3$ s^{-1} - $3.85E-3$ s^{-1}
protein degradation rate	$1.93E-4$ nM.s^{-1} - $2.89E-3$ s^{-1}
k_A	$1E-10$M - $5E-8$ M
Hill co-efficient	1 - 2. Fractions allowed.
maximum transcription rate	0.00083 nM.s^{-1} - 1 nM.s^{-1}
Unrepressed transcription rate	0.00083 nM.s^{-1} - 1 nM.s^{-1}

Table S1: Network parameter ranges

Parameter	Value	Units
Translation rate (k_{tl})	$1E-3$	s^{-1}
mRNA degradation rate (δ_m)	$2.5E-3$	s^{-1}
Protein degradation rate (δ_X)	$4E-4$	s^{-1}
Hill co-efficient (N)	1.2	scalar
k_A for h^+	$\sim 4.6E-9$	M
k_A for h^-	$\sim 4.6E-9$	M
Maximum transcription rate (P1 and P2) (β)	$4E-10$	M.s^{-1}
Unrepressed transcription rate (P3-P6) (P_{tc})	$4E-10$	M.s^{-1}

Table S2: Network parameters with equal binding strength for activators and repressors. Exact values for both activator and repressor k_A are $\sqrt[1.2]{1E-10}$.

[2] Bernstein JA, Khodursky AB, Lin PH, Lin-Chao S, Cohen SN (2002) Global analysis of mRNA decay and abundance in Escherichia coli at single-gene resolution using two-color fluorescent DNA microarrays. Proc Natl Acad Sci USA 99: 9697–702.

[3] Elowitz MB, Leibler S (2000) A synthetic oscillatory network of transcriptional regulators. Nature 403: 335–8.

[4] Lewis LK, Harlow GR, Gregg-Jolly LA, Mount DW (1994) Identification of high affinity binding sites for lexa which define new dna damage-inducible genes in Escherichia coli. Journal of Molecular Biology 241: 507–23.

[5] Liu YC, Matthews KS (1993) Dependence of trp repressor-operator affinity, stoichiometry, and apparent cooperativity on dna sequence and size. J Biol Chem 268: 23239–49.

[6] Lawrenson ID, Stockley PG (2004) Kinetic analysis of operator binding by the E. coli methionine repressor highlights the role(s) of electrostatic interactions. Febs Lett 564: 136–42.

[7] Lutz R, Lozinski T, Ellinger T, Bujard H (2001) Dissecting the functional program of Escherichia coli promoters: the combined mode of action of Lac repressor and AraC activator. Nucleic acids research 29: 3873–81.
