# Supplementary material for: A Multi-Functional Synthetic Gene Network: A Frequency Multiplier, Oscillator and Switch
Source: PLoS One. 2011 Feb 17;6(2):e16140. doi: 10.1371/journal.pone.0016140 (PMC3040778; doi:10.1371/journal.pone.0016140)
Supplement: File S3 — Frequency multiplication for an oscillating input returning to zero. (PDF) [file pone.0016140.s003.pdf]

Supporting Information S3

Frequency multiplication for an oscillating input returning to zero

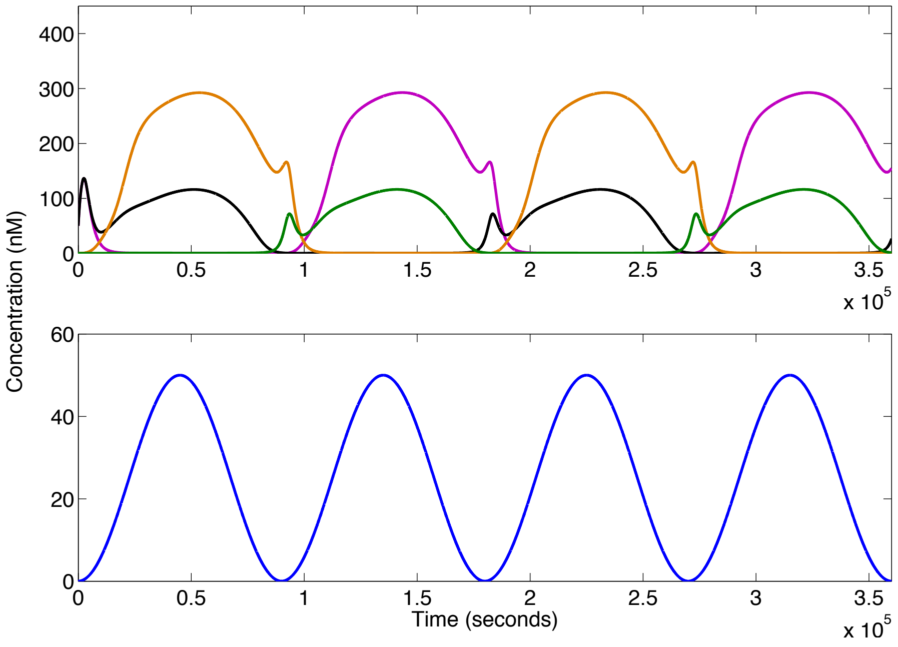

Figure S1: Frequency multiplication for an oscillating input returning to zero. Time series for the repressors R1-R4 and the input are shown in the top and bottom panels respectively. The concentrations of R1, R2, R3 and R4 are represented by pink, black, orange and green lines respectively. Initial conditions: $R1 = R2 = 50$ nM, $R3 = R4 = 0$ nM. The input is the following function: $[I](t) = -acos(Pt) + a$, where $P = \frac{2\pi}{p}$. p is the period, t is time and a is amplitude. Parameters from table 1 (main text) are used.
