# Supplementary material for: A Multi-Functional Synthetic Gene Network: A Frequency Multiplier, Oscillator and Switch
Source: PLoS One. 2011 Feb 17;6(2):e16140. doi: 10.1371/journal.pone.0016140 (PMC3040778; doi:10.1371/journal.pone.0016140)
Supplement: File S4 — Continuation of limit cycles. (PDF) [file pone.0016140.s004.pdf]

Supporting Information S4

Continuation of limit cycles

To perform the continuations in AUTO we extended the model given in the main text with an auxiliary nonlinear model uncoupled from the system which generates the required oscillatory input.

$$\dot{x} = x + \beta y - x(x^2 + y^2) \tag{1}$$
$$\dot{y} = -\beta x + y - y(x^2 + y^2) \tag{2}$$

where y has the asymptotically stable solution $y = cos(\beta t)$. The auxiliary input is used by replacing $[I]$ in the model by $-ay + b$. where $b = a + c$, a is amplitude and $c << a$ is required to ensure $-ay + b$ does not become negative, which can cause state variables to become complex, preventing continuation. $\beta = \frac{2\pi}{p}$ where p is the period. For continuations presented in the main text, $c = 1E - 9$.
