# Supplementary material for: A Multi-Functional Synthetic Gene Network: A Frequency Multiplier, Oscillator and Switch
Source: PLoS One. 2011 Feb 17;6(2):e16140. doi: 10.1371/journal.pone.0016140 (PMC3040778; doi:10.1371/journal.pone.0016140)
Supplement: File S5 — Simulations investigating parameter asymmetry. (PDF) [file pone.0016140.s005.pdf]

Supporting Information S5

Simulations investigating parameter asymmetry

Asymmetry was investigated using numerical simulations by changing one parameter in a set (e.g. changing one rate from the set of protein degradation rates) from its value in table 1 (main text), while keeping all other parameters as per table 1 (main text). Here we report the results of a representative subset of the simulations run. It should be noted that as the binding affinities of the transcriptional regulators has been represented as a k_d where $k_d = k_A^N$, changes in the Hill coefficient also induce changes in the k_A.

The effect of asymmetry on the frequency multiplier function was examined first. To test the effect of asymmetry in the intrinsic strength of the promoters we increased the unrepressed transcription rate from promoter P3 by 50% (figure S6). To test the effect of asymmetry in the binding of the "input" transcriptional activator to promoters P1 and P2 we increased the k_d for the interaction of the activator with promoter P1 by 20% ($k_d = k_A^N$ where N is the relevant Hill coefficient) (data not shown). To test the effect of asymmetry in the binding of repressors to the various promoters we increased the k_d for the interaction of repressor R2 with promoter P1 by 10%. (data not shown) To test the effect of asymmetry in the Hill coefficients we conducted separate simulations in which the Hill coefficient describing the binding of either repressor R2 or the activator to promoter P1 was changed to either 1.2 (figure S7) or 1.4 (data not shown). The frequency multiplication behaviour was observed in the presence of the tested asymmetries in intrinsic promoter strength and activator k_d values, but was not observed in the simulations with the stated asymmetries in repressor k_d values or activator/repressor Hill coefficients.

We found the oscillator function was robust to several forms of asymmetry. For example, oscillator function was observed with a 4-fold reduction in the maximum transcription rate (β) of promoter P1 (figure S8), a 4-fold increase in the k_d for the interaction of the activator with promoter P1 (data not shown), a 20% increase in the k_d for the interaction of repressor R2 with promoter P1 (data not shown), and changes in the Hill coefficient describing the binding of repressor R2 (figure S9) or the activator (data not shown) to promoter P1 to 1.4 or 1.2, respectively.

We found that the switch function was retained when the k_d for the interaction of repressor R2 with promoter P1 was increased by 50% (figure S10), but that switch function was lost when the Hill coefficient describing the binding of repressor R2 or the activator to promoter P1 was altered to 1.4 (data not shown) or 1.2 (figure S11).

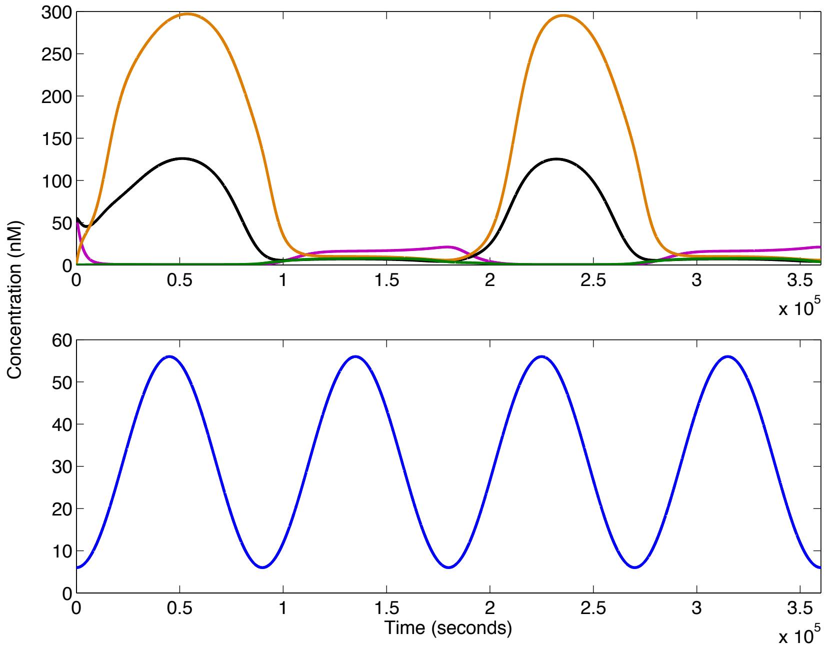

Figure S1: Frequency multiplication with 50% increase in the unrepressed rate of transcription of promoter P3 ($P3_{tc} = 6E-10$). Table 1 (main text) parameters otherwise used.

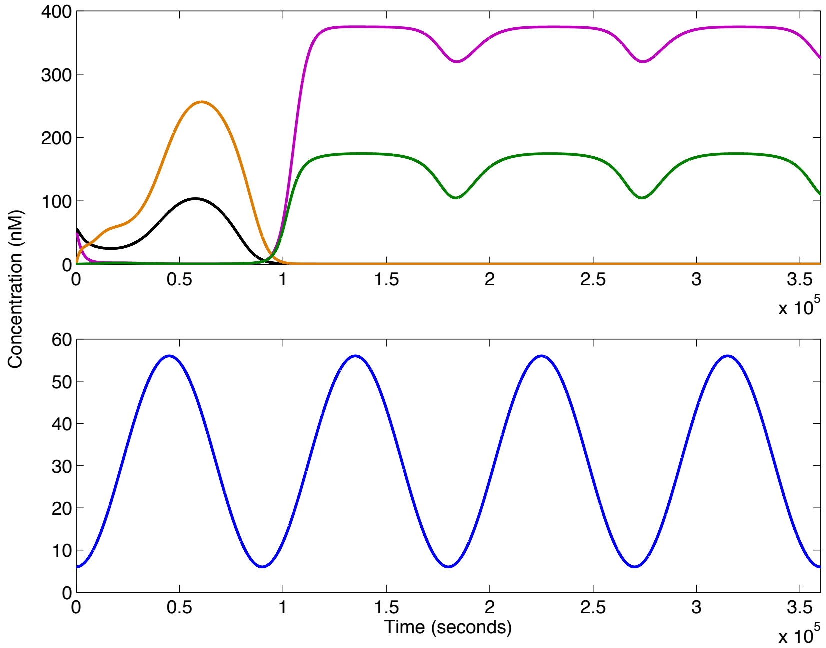

Figure S2: Failure of frequency multiplication function with Hill-coefficient describing the action of the activator regulating transcription from P1 changed to 1.2 ($N_{aP1} = 1.2$). Table 1 (main text) parameters otherwise used.

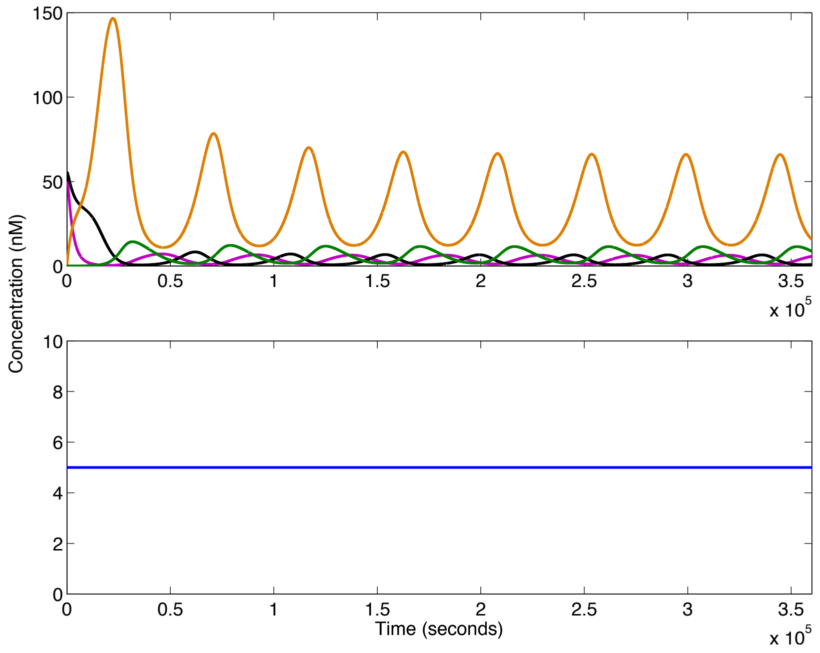

Figure S3: Oscillations with a 4-fold reduction in the maximum transcription rate of P1 (β_{P1}). Table 1 (main text) parameters otherwise used.

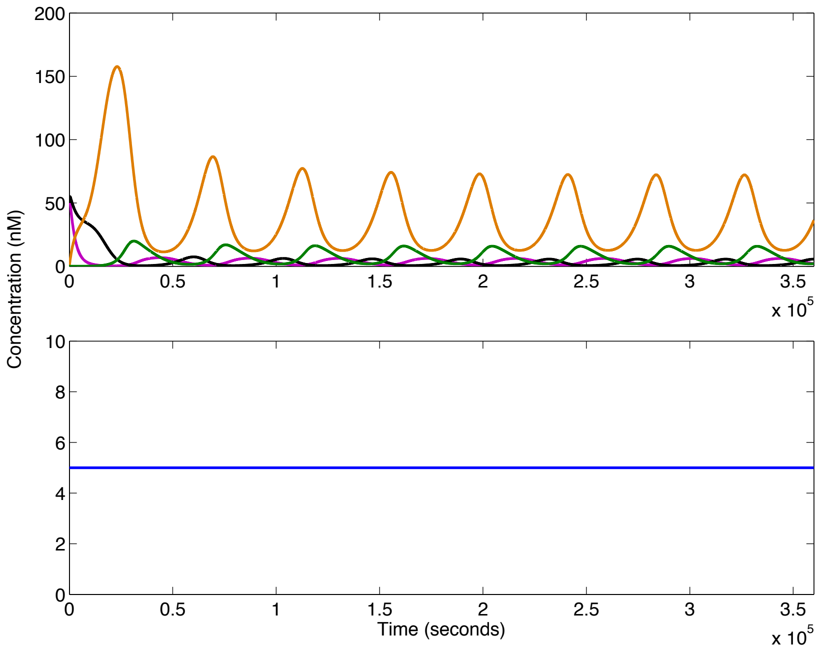

Figure S4: Oscillations with Hill-coefficient describing the action of the repressor regulating transcription from P1 changed to 1.2 ($N_{rP1} = 1.2$). Table 1 (main text) parameters otherwise used.

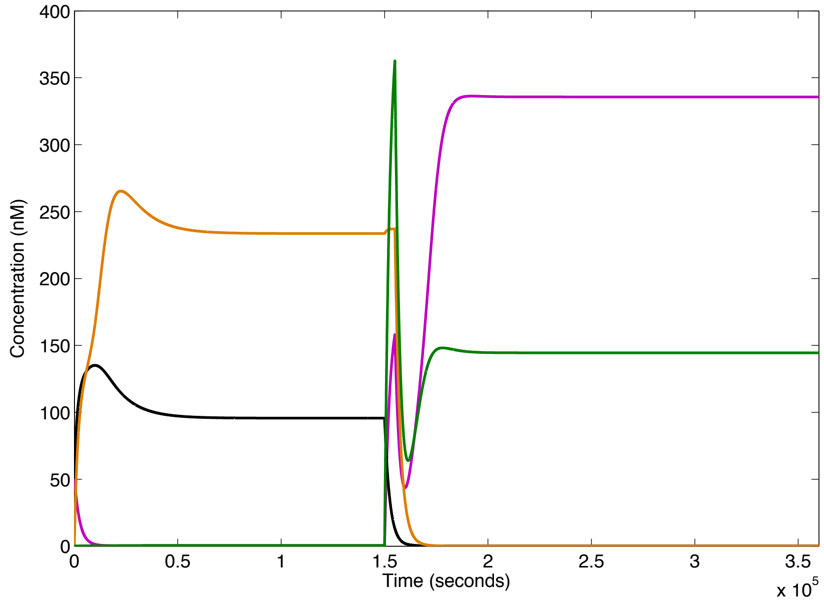

(a) Switching from (R2 & R3 high, R1 & R4 low) to (R1 & R4 high, R2 & R3 low), at an input of 50 nM.

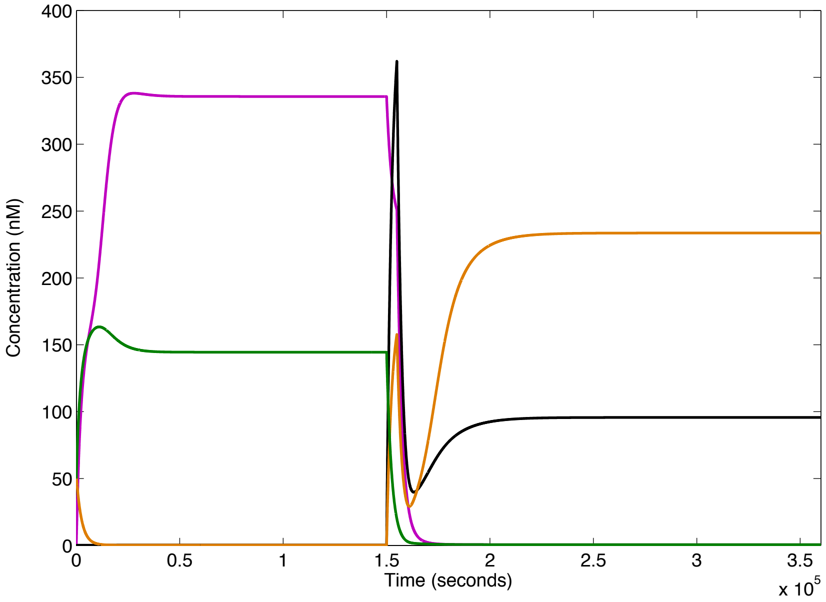

(b) Switching from (R1 & R4 high, R2 & R3 low) to (R2 & R3 high, R1 & R4 low), at an input of 50 nM.

Figure S5: Switching maintained with with the k_d of the repressor regulating promoter P1 increased by 50% with ($k_{rP1} = 1.5E - 12$) and without ($k_{rP1} = 1.5E - 7$) inducer present. Table 1 (main text) parameters otherwise used.

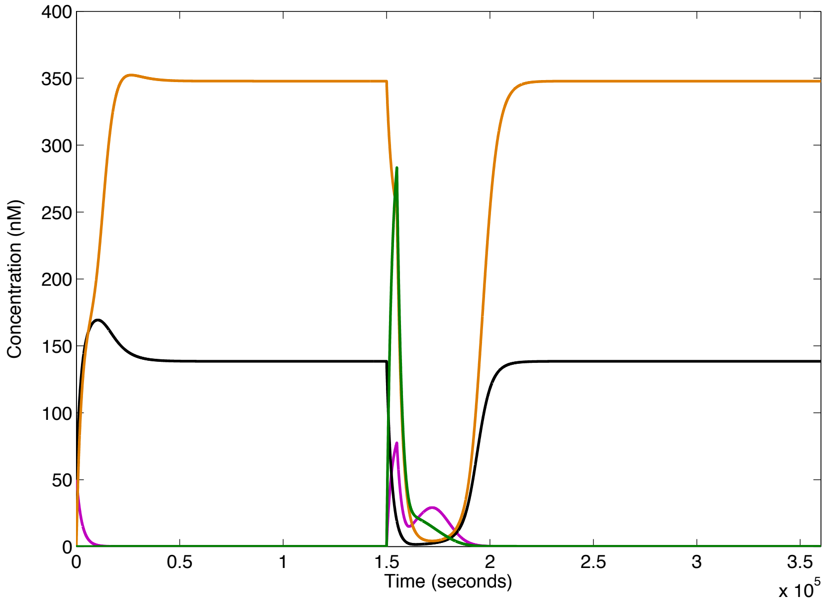

(a) Failed switching from (R2 & R3 high, R1 & R4 low) to (R1 & R4 high, R2 & R3 low), at an input of 50 nM.

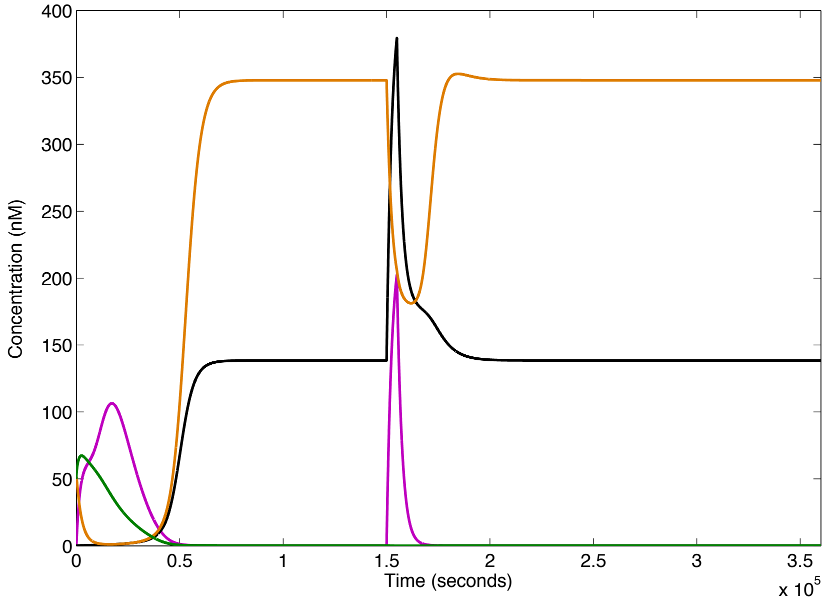

(b) Failed switching from (R1 & R4 high, R2 & R3 low) to (R2 & R3 high, R1 & R4 low), at an input of 50 nM.

Figure S6: Switching failing with the Hill-coefficient describing the action of the activator regulating transcription from P1 changed to 1.4 ($N_{aP1} = 1.4$). Table 1 (main text) parameters otherwise used.
