# Supplementary material for: A Multi-Functional Synthetic Gene Network: A Frequency Multiplier, Oscillator and Switch
Source: PLoS One. 2011 Feb 17;6(2):e16140. doi: 10.1371/journal.pone.0016140 (PMC3040778; doi:10.1371/journal.pone.0016140)
Supplement: File S6 — Stochastic simulations. (PDF) [file pone.0016140.s006.pdf]

Supporting Information S6

Stochastic Simulations

The Chemical Langevin Equations (CLEs) used were:

$$\frac{d[R1]}{dt} = a_1 + a_2 - a_3 + \frac{1}{\sqrt{V}} (\sqrt{a_1}\eta(t) + \sqrt{a_2}\eta(t) - \sqrt{a_3}\eta(t))$$
$$\frac{d[R2]}{dt} = b_1 + b_2 - b_3 + \frac{1}{\sqrt{V}} \left(\sqrt{b_1}\eta(t) + \sqrt{b_2}\eta(t) - \sqrt{b_3}\eta(t)\right)$$
$$\frac{d[R3]}{dt} = c_1 + c_2 - c_3 + \frac{1}{\sqrt{V}} (\sqrt{c_1}\eta(t) + \sqrt{c_2}\eta(t) - \sqrt{c_3}\eta(t))$$
$$\frac{d[R4]}{dt} = d_1 + d_2 - d_3 + \frac{1}{\sqrt{V}} \left(\sqrt{d_1}\eta(t) + \sqrt{d_2}\eta(t) - \sqrt{d_3}\eta(t)\right)$$

where:

$$a_1 \equiv \frac{k_{tl}\beta_{P1}}{\delta_{m14,1}}\left(\frac{[I]^{N_{aP1}}}{k_{aP1}+[I]^{N_{aP1}}}\right)\left(\frac{1}{1+\left(\frac{[R2]^{N_{rP1}}}{k_{rP1}}\right)}\right)$$

$$a_2 \equiv \frac{k_{tl}P3_{tc}}{\delta_{m1,3}}\left(\frac{1}{1+\left(\frac{[R3]^{N_{rP3}}}{k_{rP3}}\right)}\right)$$

$$a_3 \equiv \delta_{R1}[R1]$$

$$b_1 \equiv \frac{k_{tl}\beta_{P2}}{\delta_{m23,2}}\left(\frac{[I]^{N_{aP2}}}{k_{aP2}+[I]^{N_{aP2}}}\right)\left(\frac{1}{1+\left(\frac{[R4]^{N_{rP2}}}{k_{rP2}}\right)}\right)$$

$$b_2 \equiv \frac{k_{tl}P4_{tc}}{\delta_{m2,4}}\left(\frac{1}{1+\left(\frac{[R3]^{N_{r3,4}}}{k_{r3,4}}\right)}\right)\left(\frac{1}{1+\left(\frac{[R4]^{N_{r4,4}}}{k_{r4,4}}\right)}\right)$$

$$b_3 \equiv \delta_{R2}[R2]$$

$$c_1 \equiv \frac{k_{tl}\beta_{P2}}{\delta_{m23,2}}\left(\frac{[I]^{N_{aP2}}}{k_{aP2}+[I]^{N_{aP2}}}\right)\left(\frac{1}{1+\left(\frac{[R4]^{N_{rP2}}}{k_{rP2}}\right)}\right)$$

$$c_2 \equiv \frac{k_{tl}P5_{tc}}{\delta_{m3,5}}\left(\frac{1}{1+\left(\frac{[R1]^{N_{rP5}}}{k_{rP5}}\right)}\right)$$

$$c_3 \equiv \delta_{R3}[R3]$$

$$d_1 \equiv \frac{k_{tl}\beta_{P1}}{\delta_{m14,1}}\left(\frac{[I]^{N_{aP1}}}{k_{aP1}+[I]^{N_{aP1}}}\right)\left(\frac{1}{1+\left(\frac{[R2]^{N_{rP1}}}{k_{rP1}}\right)}\right)$$

$$d_2 \equiv \frac{k_{tl}P6_{tc}}{\delta_{m4,6}}\left(\frac{1}{1+\left(\frac{[R1]^{N_{r1,6}}}{k_{r1,6}}\right)}\right)\left(\frac{1}{1+\left(\frac{[R2]^{N_{r2,6}}}{k_{r2,6}}\right)}\right)$$

$$d_3 \equiv \delta_{R4}[R4]$$

$\eta(t)$ are Gaussian noise ($\mathcal{N}(0,1)$) and $V = 10^9$ is the conversion factor from molecule number to concentration.

Figures S1, S2, S3 and S4 demonstrate the network functioning as a frequency multiplier (figure S1), oscillator (figure S2), and switch (figures S3 and S4) respectively.

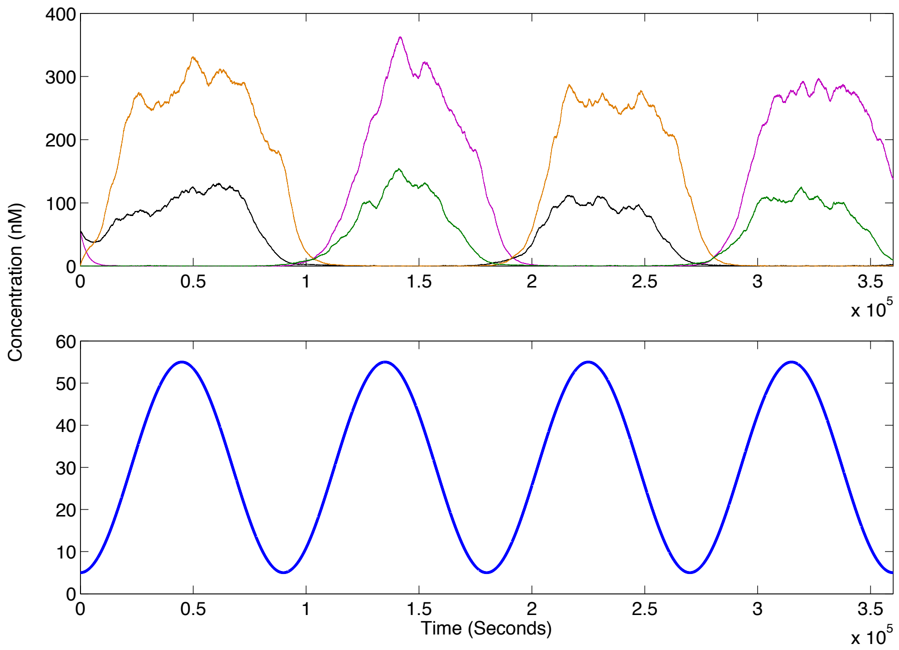

Figure S1: Effect of noise on frequency multiplier function. The graph shows CLE simulations using table 1 (main text) parameters.

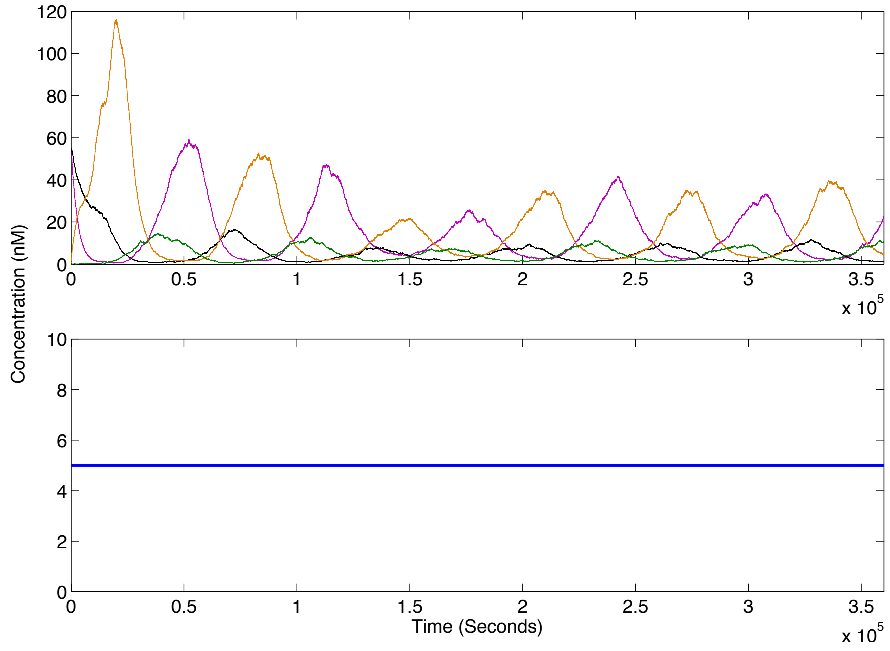

Figure S2: Effect of noise on oscillator function. The graph shows CLE simulations using table 1 (main text) parameters.

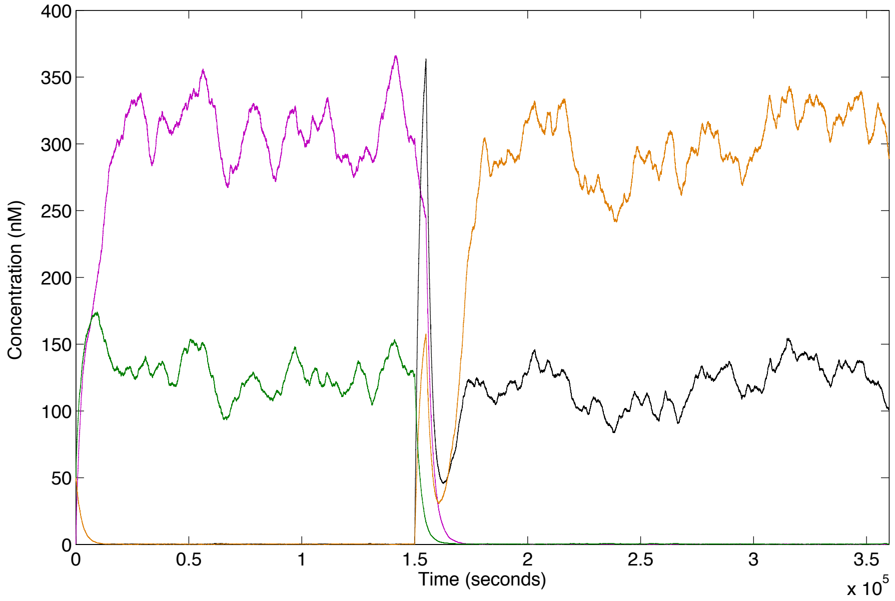

Figure S3: Effect of noise on switching function. The graph shows CLE simulations of switching from [R1 & R4 high, R2 & R3 low] to [R2 & R3 high, R1 & R4 low], using table 1 (main text) parameters

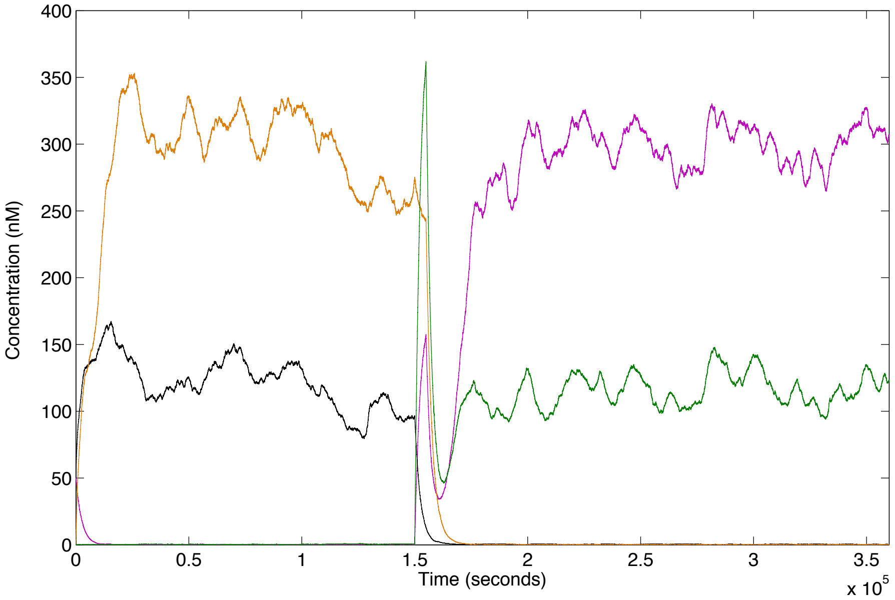

Figure S4: Effect of noise on switching function. The graph shows CLE simulations of switching from [R2 & R3 high, R1 & R4 low] to [R1 & R4 high, R2 & R3 low], using table 1 (main text) parameters.
